# Low prevalence of IgA anti-transglutaminase 1, 2, and 3 autoantibodies in children with atopic dermatitis

**DOI:** 10.1186/1756-0500-7-310

**Published:** 2014-05-22

**Authors:** Krista Ress, Kaupo Teesalu, Triine Annus, Urve Putnik, Kristi Lepik, Katrin Luts, Oivi Uibo, Raivo Uibo

**Affiliations:** 1 Department of Immunology, Institute of Bio- and Translational Medicine and Centre of Excellence for Translational Medicine, University of Tartu, Ravila 19, 50411 Tartu, Estonia; 2 Tallinn Children’s Hospital, Tervise 28, 13419 Tallinn, Estonia; 3 Department of Pediatrics, University of Tartu, Lunini 6, 51014 Tartu, Estonia; 4 Children’s Clinic, Tartu University Hospital, Lunini 6, 51014 Tartu, Estonia

**Keywords:** Autoantibodies, Atopic dermatitis, Coeliac disease, Transglutaminase 1, Transglutaminase 2, Transglutaminase 3

## Abstract

**Background:**

Atopic dermatitis (AD) is a multifactorial chronic inflammatory skin disease presenting with a relapsing clinical pattern similar to chronic autoimmune disease. Several human transglutaminases have been defined and keratinocyte transglutaminase (TG1) and epidermal transglutaminase (TG3) expressed in the epidermis are associated with epidermal barrier dysfunction. Since impairments to the epidermal barrier represent an important factor in AD, we hypothesized that IgA autoantibodies specific for TG1 (IgA-anti-TG1) and TG3 (IgA-anti-TG3) may affect AD development during childhood.

**Methods:**

Active AD patients (n = 304), 28 patients with biopsy-confirmed coeliac disease (CD), 5 patients with active AD and CD, and 55 control patients without CD and skin diseases were enrolled into the study. IgA-anti-TG1 and IgA-anti-TG3 reactivity was determined using an enzyme-linked immunosorbent assay. IgA-anti-TG2 were defined using a fluoroenzyme immunoassay.

**Results:**

IgA-anti-TG1 antibodies were found in 2% and IgA-anti-TG3 antibodies in 3% of patients with active AD. Two out of the 5 patients with AD and concomitant CD had IgA-anti-TG1 and IgA-anti-TG2 antibodies. In CD patients, 36% of individuals presented with elevated IgA-anti-TG1 antibodies and 18% presented with elevated IgA-anti-TG3 antibodies and all CD patients presented with IgA-anti-TG2 antibodies (significantly different from AD patients and controls, p < 0.05). In CD patients, IgA-anti-TG1 and/or IgA-anti-TG3 seropositivity tended to appear concurrently, whereas only one patient with AD had both types of autoantibodies.

**Conclusions:**

IgA-anti-TG1 and IgA-anti-TG3 seropositivity was rare in active AD but frequent in CD patients. The level of circulating antibodies related to skin lesions could be studied by determining the levels of IgA-anti-TG1 and IgA-anti-TG3 in skin biopsies of AD patients.

## Background

Atopic dermatitis (AD) is a multifactorial, chronic, inflammatory skin disease characterized by intense pruritus and relapsing eczema. At least 4 different factors are involved in AD progression: congenital skin barrier defects, allergy, microbial colonization, and/or autoimmunity [1]. Almost 2/3 of children that present with the clinical phenotype of AD have no identifiable allergen-specific sensitization. On the other hand, AD presents as a relapsing-remitting disease similar to that of chronic autoimmune diseases [2]. However, the role of autoimmunity in the development of AD skin lesions is not well defined despite extensive studies focusing on structural changes to the epidermis and immune dysregulation [3-6].

Recent studies have suggested a role for epidermal transglutaminases that can affect epidermal barrier dysfunction and present at higher concentrations in the skin of AD patients, especially in skin lesions, and during skin barrier repair [7-9]. Transglutaminases are a family of calcium-dependent enzymes important to various biological processes, including cell structure organization and apoptosis. Several transglutaminases have been described in humans and 4 localize to the epidermis [7,10]. Keratinocyte transglutaminase (TG1) and epidermal transglutaminase (TG3) are expressed in the spinous and granular layers of the epidermis [10], while tissue transglutaminase (TG2) is widely expressed in various tissues, including the gut epithelium. However, their expression in the epidermis can be demonstrated only under specific conditions [10,11]. TG2 was identified in 1997 as an autoantigen in coeliac disease (CD), an immune mediated gluten enteropathy with a wide range of clinical presentations [12]. IgA autoantibodies specific for TG2 (IgA-anti-TG2) are often found in patients with CD and can be found in some dermatitis herpetiformis (DH) patients. Despite the diagnostic sensitivity of IgA-anti-TG2 for CD, children < 24 months of age have been shown to have a decreased ability to produce antibodies to TG2 [13]. Instead, these patients may have antibodies against deamidated gliadin peptides (DGP), another type of autoantibody associated with CD. These antibodies develop following the deamidation of the cereal protein component gliadin by TG2 in the gut mucosa [14]. Moreover, in patients with DH, IgA autoantibodies develop against TG3, and precipitate as immune complexes in the papillary dermis thereby impacting skin lesion pathogenesis in DH patients [15,16]. Mutations in the TG1 coding gene have been reported to be deficient in lamellar ichthyosis, a disease with severely impaired epidermal barriers [10].

Since dysfunction of the epidermal barrier and autoimmunity play important roles in the pathogenesis of AD, we hypothesized that IgA autoantibodies specific for TG1 (IgA-anti-TG1) and TG3 (IgA-anti-TG3) may play a role in childhood AD development. To test this hypothesis we measured serum IgA-anti-TG1, IgA-anti-TG3, and IgA-anti-TG2 in children with active AD, in children with active AD and concomitant CD, in children with known CD, and in children with a normal small bowel mucosa without skin disease.

## Methods

### Study population

We tested 392 serum samples obtained from four groups of children: 304 patients with active AD (mean age 5.5 years, 174 boys), 5 patients with active AD and concomitant CD (mean age 5.6 years, 1 boy), 28 patients with only CD (mean age 6.2 years, 10 boys), and 55 control patients with normal small bowel mucosa without skin diseases (mean age 9.2 years, 27 boys). Patients with active AD, patients with active AD and concomitant CD, and children with normal small bowel mucosa were recruited from the Tallinn Children’s Hospital. Children with CD were studied either at Tallinn Children’s Hospital or at the Children’s Clinic of Tartu University Hospital. Patients with normal small bowel mucosa and CD were identified following histological analysis of small bowel mucosa biopsy specimens and characterized according to the European Society for Pediatric Gastroenterology, Hepatology and Nutrition (ESPGHAN) diagnostic criteria [17] taking into account Marsh classification [18]. The clinical presentation of CD was defined as classical, atypical gastrointestinal, extraintestinal, or silent type [14,19]. No patients in the study were on a gluten-free diet or systemic immunomodulatory treatment.

Written informed consent was obtained from all study participants or their parents or legal guardians. The study was conducted in accordance with the ethical guidelines established by the Declaration of Helsinki and approved by the Ethics Review Committee on Human Research of the University of Tartu, Estonia.

### Total IgA, IgA-anti-TG2, and IgA-anti-DGP

To exclude an IgA deficiency, total serum IgA was determined for all samples using a chemiluminescence assay (Roche Diagnostics, Burgess Hill, England) and the results were compared to age-specific reference values. The IgA-anti-TG2 and IgA-anti-DGP responses were measured using a fluoroenzyme immunoassay using the ImmunoCAP EliA Celikey system (Thermo Fisher Scientific, Uppsala, Sweden). According to the manufacturer’s recommendations, IgA-anti-TG2 and IgA-anti-DGP values of 10 EliA U/ml or higher were considered positive, and values lower than 7 EliA U/ml were considered negative. Borderline values (between 7 and 10 EliA U/ml) were considered negative for the purposes of carrying out statistical analysis.

### IgA-anti-TG1 and IgA-anti-TG3

The IgA-anti-TG1 and IgA-anti-TG3 were measured by an enzyme-linked immunosorbent assay as described earlier (ELISA) [20] using recombinant TG1 and TG3 as target antigens (Zedira GmbH, Darmstadt, Germany). Briefly, universal binding 96-well microtiter plates (Thermo Fisher Scientific OY, Vantaa, Finland) were coated with 0.5 μg TG1 or TG3 per well overnight at 4°C. After washing and rinsing of the wells with 5% sucrose, plates were dried and kept at 4°C until use. Serum samples were diluted 1:100 in TBS-T buffer (25 mM Tris–HCl, 150 mM NaCl, 0.1% Tween 20, pH 7.4) and incubated in duplicate wells for 1 h at room temperature. After washing with TBS-T 5 times, wells were incubated for 30 min with a 1:1000 dilution (in TBS-T) of alkaline phosphatase (AP)-conjugated goat anti-human IgA (Invitrogen Corporation, Camarillo, USA). Reactivity was visualized by developing using the substrate 4-p-nitrophenyl phosphate for 30 min and measuring absorbance values at 405 nm with a 492 nm subtraction. Antibody levels were expressed in arbitrary units (AU) as percentages of the reference serum OD values. The assay cut-off values for IgA-anti-TG1 and IgA-anti-TG3 were calculated by determining the mean AU + 2SD in the control subjects, which yielded IgA-anti-TG1 values higher than 37.3 AU and IgA-anti-TG3 values higher than 48.8.

### Statistical analysis

The data were expressed as absolute numbers or proportions for categorical variables and as means for continuous variables. The diagnostic performance of both assays in terms of sensitivity and specificity, expressed as a percentage, was calculated based on the cut-off values described above. For statistical analyses, the R software for Windows (The R Foundation for Statistical Computing, Vienna, Austria) and the MedCalc statistical software (MedCalc Software, Mariakerke, Belgium) were used. Differences between subgroups were analyzed using the Fisher’s exact test or the Wilcoxon rank sum test as appropriate. A p-value ≤0.05 was considered significant.

## Results

### Determination of total IgA, IgA-anti-TG2, and IgA-anti-DGP

Seven (2%) of the AD patients and three (5%) control patients were IgA deficient and therefore excluded from further antibody and statistical analysis. Differences in IgA deficiency rates between these groups were not statistically significant.

Seropositivity rates of the different study groups are shown in Table 1. No patients in the AD group presented with IgA-anti-TG2 and 7 patients (2%) had slight IgA-anti-DGP reactivity (all were >2 years of age and none were seropositive for other markers). Of the 5 AD patients with concomitant CD, 4 had IgA-anti-TG2 and 3 had IgA-anti-DGP antibodies. In CD patients IgA-anti-TG2 was identified in 27 (96%) and IgA-anti-DGP in 24 (86%, all IgA-anti-TG2 positive). In the control group, only 1 patient presented with borderline IgA-anti-TG2 values without any accompanying seropositivity. Elevated IgA-anti-DGP values were found in the sera of 2 control group patients, both <2 years of age and without other detectable autoantibodies. No changes to the small intestine mucosa were identified in these patients.

**Table 1:** Seropositivity rates of the different patient groups*

	**IgA-anti-TG1**		**IgA-anti-TG2**		**IgA-anti-TG3**		**IgA-anti-DGP**	
	**+**	**-**	**+**	**-**	**+**	**-**	**+**	**-**
**AD**	6	291	0	297	9	288	7	290
**(n = 297)**	(2%)	(98%)		(100%)	(3%)	(97%)	(2%)	(98%)
**AD + CD**	2	3	4	1	0	5	3	2
**(n = 5)**	(40%)	(60%)	(80%)	(20%)		(100%)	(60%)	(40%)
**CD**	10	18	27	1	5	23	24	4
**(n = 28)**	(36%)	(64%)	(96%)	(4%)	(18%)	(82%)	(86%)	(14%)
**Controls**	2	50	1	51	2	50	2	50
**(n = 52)**	(4%)	(96%)	(2%)	(98%)	(4%)	(96%)	(4%)	(96%)
	AD vs controls p = 0.340		AD vs controls p = 0.14		AD vs controls p = 0.671		AD vs controls p = 0.628	
	AD vs CD p = 0**		AD vs CD **p = 0**		AD vs CD **p < 0.005**		AD vs CD **p = 0**	
	AD vs ADCD **p = 0.006**		AD vs ADCD **p = 0**		AD vs ADCD p = 1		AD vs ADCD **p < 0.005**	
	ADCD vs controls **p = 0.035**		ADCD vs controls **p < 0.005**		ADCD vs controls p = 1		ADCD vs controls **p < 0.005**	
	ADCD vs CD p = 1		ADCD vs CD p = 0.284		ADCD vs CD p = 0.569		ADCD vs CD p = 0.216	
	CD vs controls **p < 0.005**		CD vs controls **p = 0**		CD vs controls **p = 0.048**		CD vs controls **p = 0**	

*Children with low serum IgA were excluded.

**p values marked in bold are statistically significant.

### IgA-anti-TG1

IgA-anti-TG1 antibodies were found in all patient groups (Table 1). Six patients with AD (2%) and 2 control patients (4%) had elevated IgA-anti-TG1, but were IgA-anti-TG2 negative. Two patients with AD also presenting with CD had IgA-anti-TG1 responses and both were also positive for IgA-anti-TG2 and IgA-anti-DGP. Ten patients (36%) with CD had IgA-anti-TG1 antibodies and all were IgA-anti-TG2 positive. The IgA-anti-TG1 positive CD patients were also IgA-anti-DGP positive except for 1 patient with borderline IgA-anti-DGP values but with marked IgA-anti-TG2 responses.

### IgA-anti-TG3

IgA-anti-TG3 antibodies were found in all patient groups except patients with concomitant AD and CD (Table 1). Elevated IgA-anti-TG3 responses were detected in 9 patients (3%) with AD and in 3 control patients (4%), none of them presented with elevated IgA-anti-TG2 levels. Five patients (18%) with CD had elevated IgA-anti-TG3 response, all of them were IgA-anti-TG2 and IgA-anti-DGP positive.

### IgA-anti-TG1 and IgA-anti-TG3 in different patient groups

**Figure 1:**
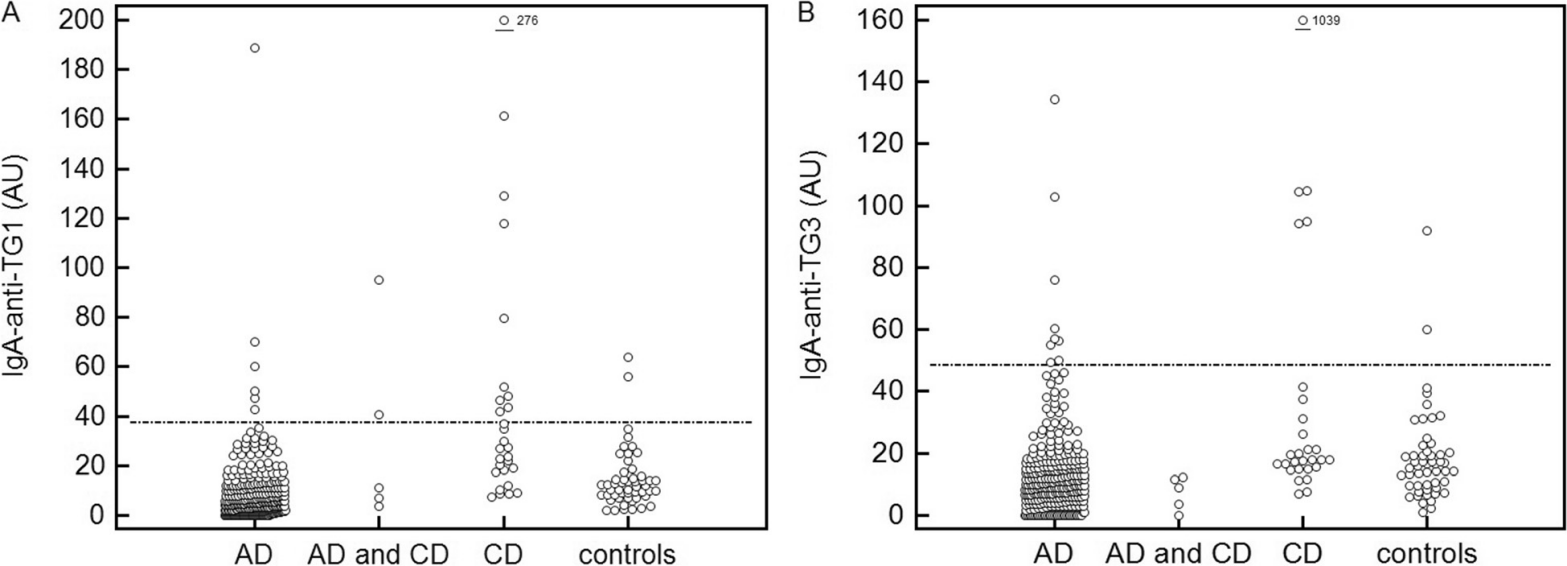
**IgA-anti-TG1 (A) and IgA-anti-TG3 (B) values in the different patient groups.** AD, atopic dermatitis; CD, coeliac disease. *Horizontal line - reference value based on the mean + 2SD of the control subjects.

Among the AD patients the IgA-anti-TG1 and IgA-anti-TG3 responses were as prevalent as responses observed in the control group. IgA-anti-TG1 and IgA-anti-TG3 responses were more common among CD patients than among controls or AD patients (p < 0.05). Among the CD patients, both the mean levels of IgA-anti-TG1 and IgA-anti-TG3 antibodies were higher compared to levels observed in AD patients or in the control group (p < 0.05) (Figure 1). In AD patients with concomitant CD, IgA-anti-TG1 responses were as prevalent as in the CD patients but more common compared to controls (p < 0.05) or AD patients (p < 0.005), indicating that IgA-anti-TG1 and IgA-anti-TG3 responses were associated with CD and not AD.

Comparisons between all seropositive cases from the different patient groups revealed that only 1 patient with active AD had both elevated IgA-anti-TG1 and IgA-anti-TG3 antibody levels, however, patients presenting with CD or with AD and concomitant CD tended to be seropositive for IgA-anti-TG1 and/or IgA-anti-TG3 together with IgA-anti-TG2 and IgA-anti-DGP seropositivity (see Additional file 1: Table S1 for more details).

**Figure 2:**
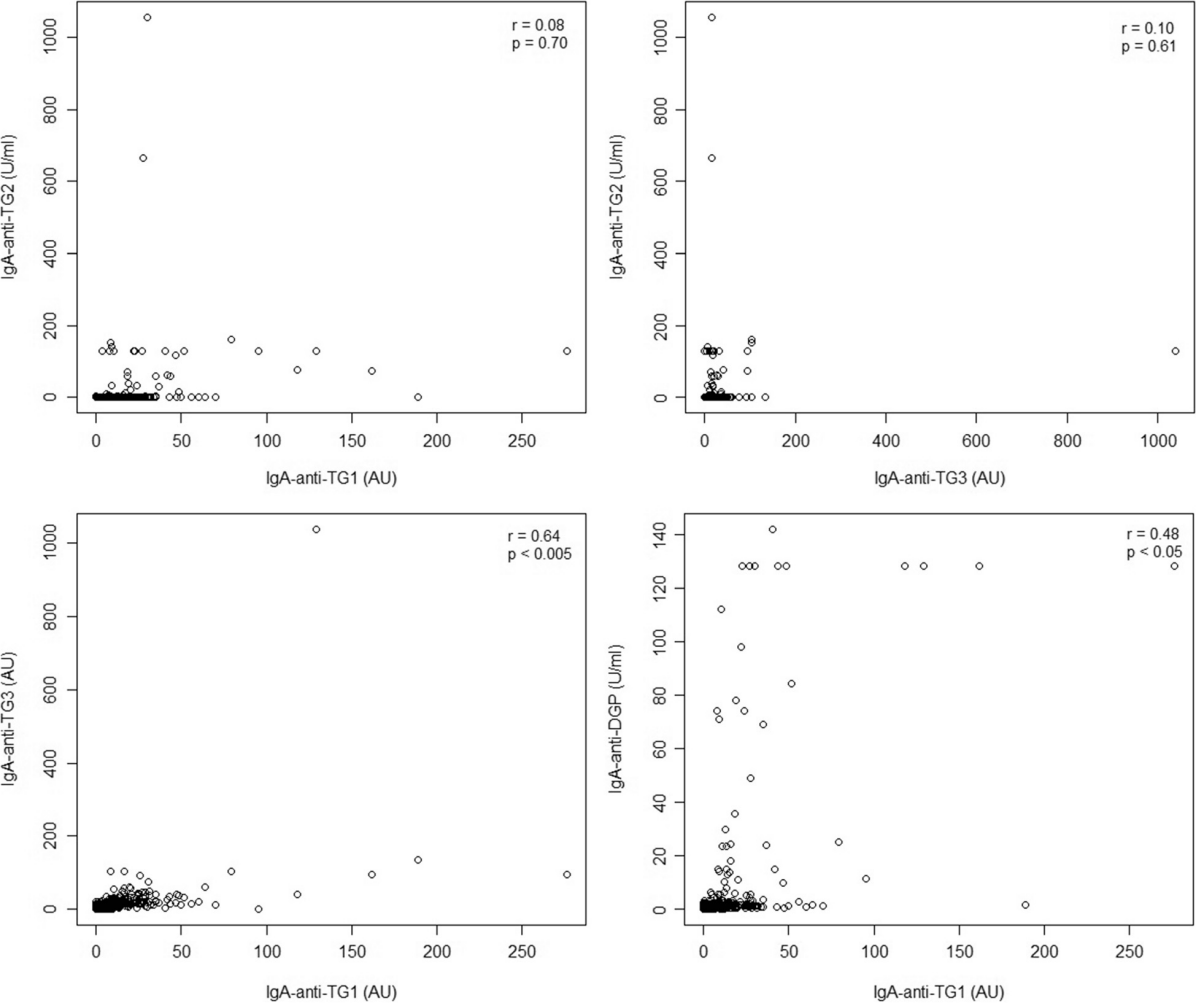
Correlation plots for IgA-anti-TG1, IgA-anti-TG2, IgA-anti-TG3 and IgA-anti-DGP in patients with CD.

Elevated IgA-anti-TG1 and IgA-anti-TG3 levels were also found in 3 control patients. One patient presented with long-lasting diarrhoea and elevated levels of both antibodies and was later diagnosed with cystic fibrosis. The other 2 patients were either IgA-anti-TG1 or IgA-anti-TG3 seropositive. However, these 2 patients presented with acute gastritis resulting from a *Helicobacter pylori* infection that may be a predisposing factor for developing IgA-anti-TG1 and/or IgA-anti-TG3 responses.When comparing IgA-anti-TG1, IgA-anti-TG2, and IgA-anti-TG3 responses using the Spearman’s rank correlation, a statistically significant correlation was noted between IgA-anti-TG1 and IgA-anti-TG2 response (r = 0.51), IgA-anti-TG3 and IgA-anti-TG2 response (r = 0.44) and between the IgA-anti-TG1 and IgA-anti-TG3 assay response (r = 0.70). When comparing antibody responses in CD patients, a statistically significant correlation was noted between the IgA-anti-TG1 and IgA-anti-TG3 response (r = 0.64) and between the IgA-anti-TG1 and IgA-anti-DGP assay response (r = 0.48) (Figure 2).

## Discussion

In recent years, characterization of skin-related immune processes and involvement of autoimmune reactions associated with the pathogenesis of AD have received much attention [6]. In the present study we determined whether TG1 and TG3 (enzymes that maintain skin barrier integrity) could be targets for IgA autoantibodies in patients with AD.

We found no increases in IgA-anti-TG1 and IgA-anti-TG3 antibodies, nor in the CD biomarkers IgA-anti-TG2 and IgA-anti-DGP among AD patients compared to the control group. In the group of AD patients with slightly elevated IgA-anti-DGP the antibody concentration was relatively low compared to the corresponding concentrations in biopsy-confirmed CD patients, therefore, a longitudinal clinical follow-up can be recommended in these children to confirm persistent seropositivity.

Our findings suggested that IgA antibodies specific for TG isoenzymes (expressed in the dermis) are not characteristic of AD. However, the levels of IgA-anti-TG1 and IgA-anti-TG3 were significantly higher in CD patients compared with patients in the other groups. IgA-anti-TG3 antibodies were found in 18% of CD patients in concordance with earlier studies where IgA-anti-TG3 antibodies were detected in 11-33% of untreated CD patients [21,22]. In the CD group, IgA-anti-TG1 and IgA-anti-TG3 responses tended to appear in parallel and all seropositive CD patients also had elevated IgA-anti-TG2 responses. Considering IgA-anti-TG3 as a marker for DH, the higher prevalence of IgA-anti-TG3 in CD patients may indicate the possible clinical development of the CD skin phenotype later in life [16,23].

Somewhat surprisingly, IgA-anti-TG1 responses were detected frequently (36%) in CD patient sera. This observation had not previously been described and is at this time difficult to explain. However, it clearly shows that IgA reactivity against other TG family members needs to be further studied in patients with CD. For example, antibodies against neuronal transglutaminase (TG6) have been described in a subgroup of patients with gluten-sensitive cerebellar ataxia [24].

The identification of autoantibodies against different types of TG does not rule out the potential for cross-reactivity between TGs. When comparing IgA-anti-TG1, IgA-anti-TG2, and IgA-anti-TG3 levels between the study groups we identified a moderate but statistically significant correlation between the IgA-anti-TG1 and IgA-anti-TG2 assays and between results of the IgA-anti-TG3 and IgA-anti-TG2 assays, indicating possible cross-reactivity between the tested TGs. However, this potential cross-reactivity does not conceal specific reactivity against various TGs that may exist since none of the controls or AD patients had significantly elevated IgA-anti-TG1 and/or IgA-anti-TG3 responses in association with IgA-anti-TG2 responses.

Data presented in this report support a role for antigen-specific IgA reactivity against dermal TGs in a minority of children. Whether these antibodies have a prognostic value in the diagnosis of autoimmune diseases will require further studies.

## Conclusions

Based on experiments designed to evaluate the levels of antibodies against TG1, TG2, and TG3, no significant association was found between any of these autoantibodies and AD. On the contrary, we showed that IgA-anti-TG1 and IgA-anti-TG3 responses occurred frequently in CD patients suggesting that circulating antibodies to skin transglutaminases TG1 and TG3 were not related to AD. Further research should focus on measuring IgA-anti-TG1 and IgA-anti-TG3 responses in skin biopsies from AD patients.

## Competing interests

The authors declare that they have no competing interests.

## Authors’ contributions

KR carried out the immunoassays, performed the statistical analysis, and participated in the preparation of the manuscript. KT participated in development of the immunoassays and in the preparation of the manuscript. TA, UP, KLe and KLu participated in recruitment of the study population and in the preparation of the manuscript. OU and RU participated in the design of the study and participated in the preparation of the manuscript. All authors read and approved the final manuscript.

## Supplementary Material

Additional file 1: Table S1 Detailed characterization of IgA-anti-TG1 and IgA-anti-TG3 seropositive cases. Click here for file
