# Supplementary material for: Low prevalence of IgA anti-transglutaminase 1, 2, and 3 autoantibodies in children with atopic dermatitis
Source: BMC Res Notes. 2014 May 22;7:310. doi: 10.1186/1756-0500-7-310 (PMC4045883; doi:10.1186/1756-0500-7-310)
Supplement: Additional file 1: Table S1 — Detailed characterization of IgA-anti-TG1 and IgA-anti-TG3 seropositive cases. [file 1756-0500-7-310-S1.pdf]

Additional table. Characterization of IgA-anti-TG1 and IgA-anti-TG3 seropositive cases.

Patient no.	Age (y)	Sex	IgA-anti-TG2 (pos >10 U/ml)	IgA-anti-TG1 (pos >37.3 AU)	IgA-anti-TG3 (pos > 48.4 AU)	IgA-anti-DGP (pos > 10 U/ml)	Clinical presentation
**Atopic dermatitis**							
AT002	10	F	0.7	15.2	**49.3***	0.3	
AT048	9	F	0.6	10.5	**55.2**	0.9	
AT052	4	F	0.1	19.7	**60.5**	0.3	
AT215	6	M	0.2	**60.0**	19.0	0.8	asthma
AT219	3	M	0.0	20.2	**56.5**	0.7	allergic rhinoconjunctivitis, asthma
AT247	18	F	0.5	**42.8**	34.4	0.9	allergic rhinoconjunctivitis, asthma
AT250	8	F	0.1	16.8	**102.8**	1.7	
AT266	3	M	0.2	**47.2**	39.6	0.5	
AT267	18	M	0.4	**188.6**	**134.6**	1.7	allergic rhinoconjunctivitis, asthma, urticaria
AT298	12	F	1.0	31.3	**50.2**	1.1	allergic rhinoconjunctivitis, asthma
AT329	2	F	0.2	16.2	**57.1**	0.3	
AT361	17	F	0.4	30.4	**75.9**	1.1	
AT380	6	M	0.1	**70.0**	12.0	1.4	
AT381	2	M	0.4	**50.0**	12.0	1.2	
**Atopic dermatitis and concomitant coeliac disease**							
AT018	7	F	**128**	**95.2**	0	**11.5**	silent coeliac disease, allergic rhinitis, asthma
AT091	3	F	**>128**	**40.5**	3.5	**142.0**	silent coeliac disease
**Coeliac disease**							
TT006	10	M	**128.0**	**51.8**	31.1	**84.1**	atypical gastrointestinal coeliac disease
CD08	10	M	**117**	**46.6**	17.1	9.9	classical coeliac disease; type 1 diabetes
CD34	9	F	**160**	**79.5**	**104.8**	25.0	classical coeliac disease; type 1 diabetes
CD45	11	M	**152**	8.7	**104.7**	15.0	silent coeliac disease
VA4	1	F	**73.9**	**161.6**	**94.3**	128.0	classical coeliac disease
VA27	7	F	**128**	**276.3**	**95.0**	128.0	classical coeliac disease; type 1 diabetes
VA28	1	F	**13.5**	**48.2**	37.5	128.0	classical coeliac disease
VA36	15	M	**128**	**129.0**	**1039.5**	128.0	classical coeliac disease
VA40	8	F	**59.2**	**43.6**	15.7	128.0	classical coeliac disease; type 1 diabetes
VA67	14	F	**60.6**	**42.0**	26.3	15.0	classical coeliac disease; type 1 diabetes
VA69	1	F	**76.2**	**117.8**	41.6	128.0	classical coeliac disease
**Control group**							
TT009	0.5	F	0.0	**63.8**	**60.2**	1.7	cystic fibrosis, diarrhoea
TT031	10	F	0.2	**55.9**	14.2	2.9	gastritis (*H. pylori*)
TT053	13	M	0.7	25.5	**91.9**	1.1	gastritis (*H. pylori*)

*Values marked in bold – positive values for IgA-anti-TG2 and IgA-anti-DGP (based on manufacturer's recommendations) and for IgA-anti-TG1 and IgA-anti-TG3 (based on the mean+2SD in control subjects).
